# A rare case of clear cell sarcoma of the foot with a cascade of pathological misdiagnosis—the importance of expert sarcoma pathology

**DOI:** 10.3332/ecancer.2022.1459

**Published:** 2022-10-31

**Authors:** Anand Rajendran, Gaurav Gupta, Adarsh Barwad, Sameer Rastogi

**Affiliations:** 1 Department of Medicine, All India Institute of Medical Sciences, New Delhi 110029, India; 2 Department of Pathology, All India Institute of Medical Sciences, New Delhi 110029, India; 3 Department of Medical Oncology, All India Institute of Medical Sciences, New Delhi 110029, India

**Keywords:** sarcoma, clear cell sarcoma, misdiagnosis, EWSR1, diagnostic discrepancy

## Abstract

Sarcoma pathology discrepancy is well known owing to the extremely heterogenous and rare nature of this tumour. Through this case, we want to highlight the difficulty that a patient has to undergo in a case of misdiagnosis. A 20-year-old male presented with swelling in the right foot for 4 months, which was initially diagnosed as alveolar rhabdomyosarcoma, subsequently as synovial sarcoma and finally as Ewing’s sarcoma (based upon positive Ewing Sarcoma Breakpoint Region 1 (*EWSR1*) by fluorescence in situ hybridisation and he underwent neoadjuvant chemotherapy and surgical excision with grafting before he presented to our institute, where the pathologists reviewed the biopsy slides, which were positive for HMB45 and negative for Melan-A suggestive of clear cell sarcoma. The next-generation sequencing suggested *EWSR1-ATF1* fusion, which again reinforced the diagnosis. This case throws light on the importance of expert pathology and interpreting molecular results in the right context.

## Introduction

Soft tissue sarcomas (STSs) are a group of remarkably diverse neoplasms that frequently pose significant diagnostic challenges to surgical pathologists [1]. Pathological discrepancy between various institutes has been reported from 25% to 50% across the globe [2–6]. Molecular testing is now becoming an integral part of diagnosis of sarcomas and thus influencing the management. In a prospective multi-centre observational study (GENSARC) which included 384 patients with sarcoma, diagnosis was modified eventually by molecular methods in 53 patients with the maximum percentage among dedifferentiated liposarcoma patient cohort (23%) [7].

Clear cell sarcoma (CCS) is a rare tumour first described in 1968 and also referred to as malignant melanoma of soft parts [8]. It has characteristics of both STS and malignant melanoma with a predilection for the deep soft tissues of lower extremity and typically affects young adults [9]. This tumour is characterised by the translocation t(12;22)(q13;q12) and consequent fusion of the Ewing Sarcoma Breakpoint Region 1 (EWSR1) and ATF1 genes which is not documented in malignant melanoma [9] and its detection using advanced molecular techniques helps in the confirmation of a diagnosis of CCS. The literature is rife with studies in which CCS has been misdiagnosed as melanoma, metastatic carcinoma, fibrosarcoma, etc. [10–13].

A thorough understanding about the molecular abnormalities of STSs is necessary to avoid misdiagnosis of a tumour, as there are multiple tumours which behave differently with similar morphology and immunopositivity. This case where the diagnosis was altered thrice before coming to the final diagnosis emphasises the need for timely referral to a sarcoma specialist to avoid misdiagnosis and mismanagement.

### Case

**Figure 1.**
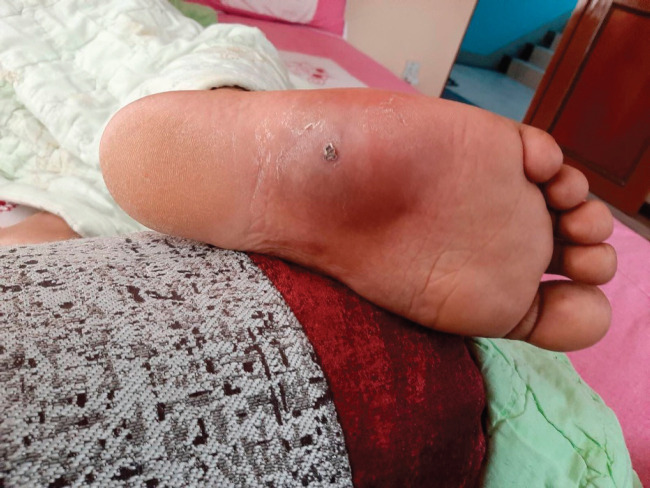
Photograph of the swelling on the plantar aspect of right foot, taken before chemotherapy, 3 months after the onset.

A 20-year-old male from Kathmandu, Nepal, developed pain on the undersurface of his right foot since January 2021, which was exaggerated by walking. He also noticed a swelling which was better felt than seen (Figure 1) at the site where he was feeling pain and after 3 months since onset of symptoms, an ultrasound of the right foot was done, which showed a suspicious mass under the skin of the sole and an MRI was advised for further characterisation.

**Figure 2.**
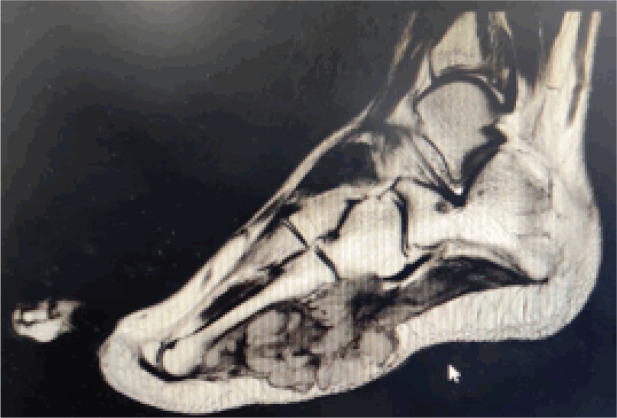
MRI image of right foot (T1W post contrast). Lobulated soft tissue mass with poorly defined margins in the lateral plantar aspect of right foot measuring approx. 4.5 × 7.5 × 3.8 cm (CC × AP × TR). Non- enhancing regions likely representing cystic/necrotic changes. Insinuating between the 4th and 5th metatarsals and involving the intrinsic muscles and abutting the lateral aspect of flexor digitorum longus/brevis muscles and tendons. Also involving the subcutaneous tissue on the plantar aspect of the foot. No definite evidence of signal abnormality or destructive changes in the bones.

An MRI was done (Figure 2) on the 24th of April, which showed a heterogeneously enhancing lobulated mass of size 4.5 × 7.5 × 3.8 cm with occasional non-enhancing areas suggestive of necrosis, involving both subcutaneous tissue and overlying muscles bones and tendons.

The patient underwent a trucut biopsy of the tumour and on histopathological examination, sheets of malignant, round to polygonal cells with intervening fibrous septa were seen with foci of necrosis. The overall impression in the initial report was alveolar rhabdomyosarcoma. Immunostains were not used at this point of time. The patient was administered neo-adjuvant chemotherapy with two cycles of vincristine, cyclophosphamide and dactinomycin on the 12th of May and the 3rd of June 2021 following which, a repeat MRI of foot was done on the 21st of June, which showed no interval changes compared to the previous MRI. Histopathology was again reviewed in another Centre and immunohistochemistry (IHC) markers were done, and the cells were positive for CD99 and TLE-1 and negative for CK, Desmin, S100, CD45, Myo-D1, TdT, NKX2.2 and synaptophysin. The report was subsequently reviewed and a diagnosis of monophasic synovial sarcoma was made. He further underwent wide local excision of the tumour and Ray’s amputation of the 4th and 5th toes of right foot was done with skin and soft tissue grafting on the 27th of June.

**Figure 3.**
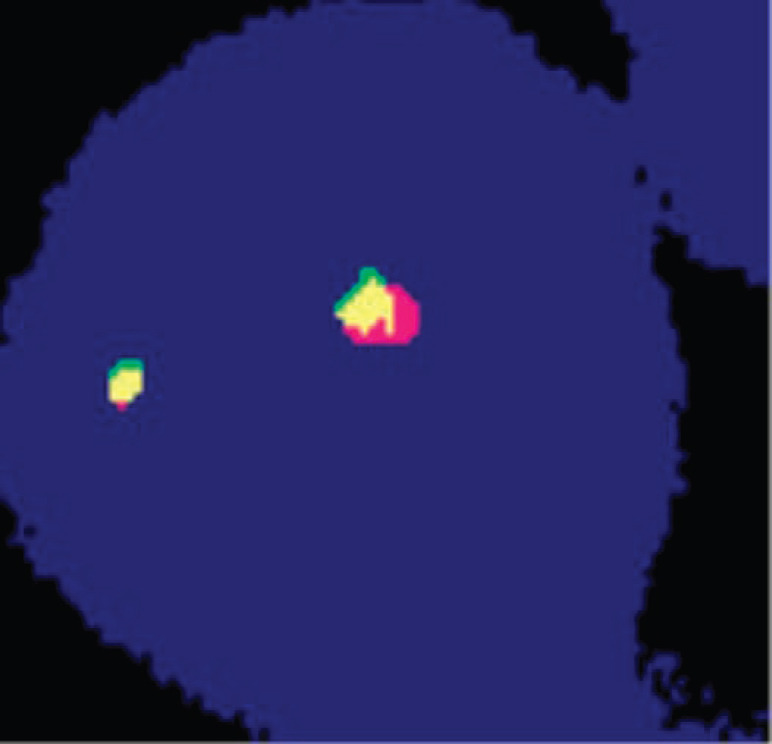
Cell showing two green-orange (fusion) signals indicating negative for SYT gene rearrangement. Test method: FISH. Probe description: Vysis LSI EWSR1 (22q12) Break Apart Rearrangement Probe (CE).

**Figure 4.**
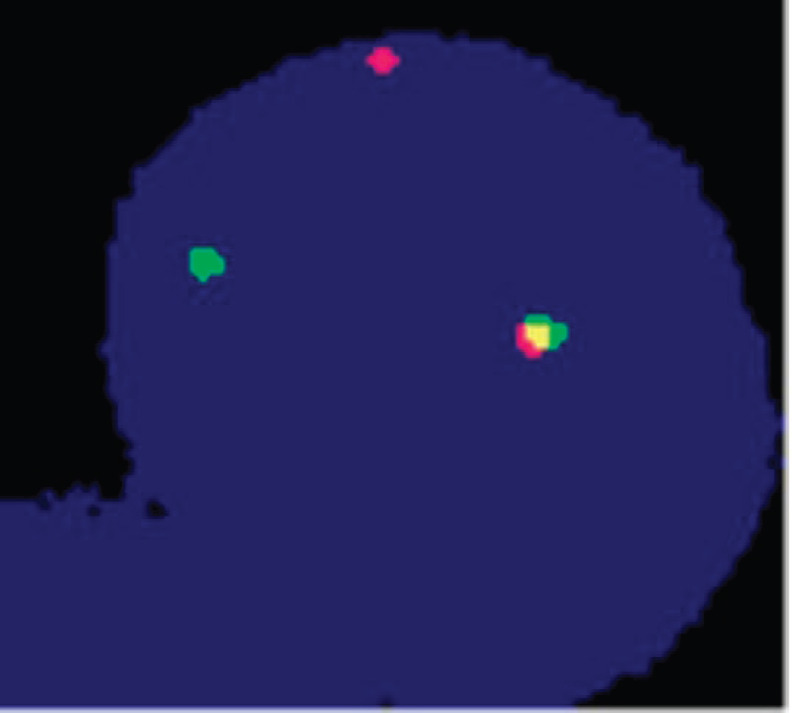
Cell showing one green-orange (yellow) fusion, one orange signal and one green signal, indicating positive for rearrangement of the EWSR1 gene. Test method: FISH. Probe description: Vysis LSI EWSR1 (22q12) Break Apart Rearrangement Probe (CE).

In the biopsy specimen, a break-apart fluorescence in situ hybridisation (FISH) for *EWSR1* and *SS18* rearrangement was done which detected *EWSR1* rearrangement (Figures 3 and 4) and hence the diagnosis was changed from synovial sarcoma to Ewing’s sarcoma/PNET.

**Figure 5.**
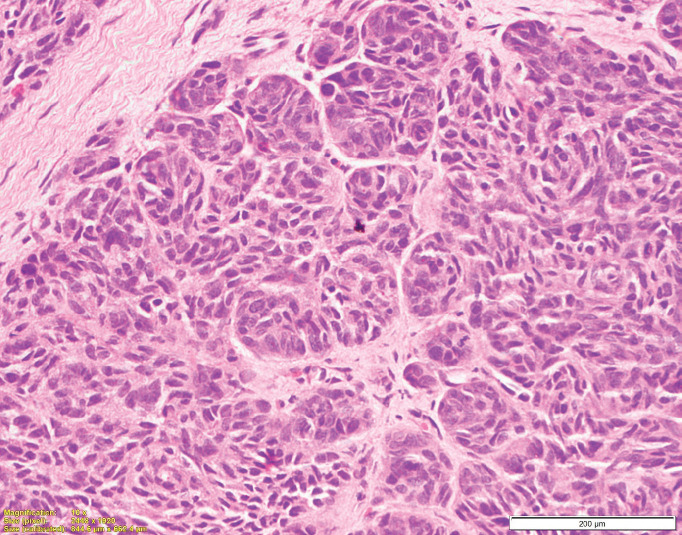
Histopathology photomicrograph of the tumour showing oval to spindle shaped cells arranged in nests as well as syncytial pattern. The tumour cells showed moderate pleomorphism with vesicular chromatin and prominent nucleoli with moderate to abundant cytoplasm with epithelioid morphology at places. (H&E 200×).

**Figure 6.**
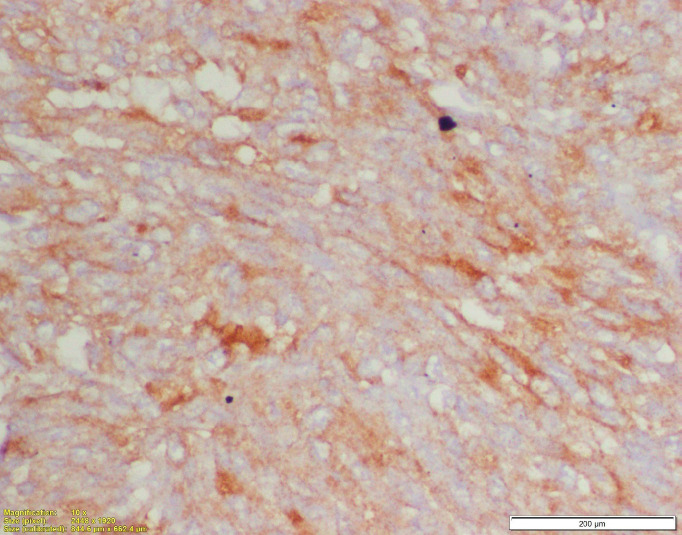
Immunohistochemistry for HMB45 showing diffuse cytoplasmic positivity in the tumour cells.

At our institute, the Pathologists reviewed the slides of the resected specimen. The morphology of the tumour showed oval to spindle shaped cells arranged in nests as well as syncytial pattern. The tumour cells showed moderate pleomorphism with vesicular chromatin and prominent nucleoli with moderate to abundant cytoplasm with epithelioid morphology at places (Figure 5). The tumour cells were immunopositive for HMB45 (Figure 6) and immunonegative for Melan A. Therefore, the *EWSR1* rearrangement detected earlier was related to CCS rather than Ewing’s sarcoma/PNET.

We further sent the specimen for next-generation sequencing analysis, which detected *EWSR1::ATF* gene fusion, which confirmed the diagnosis of CCS. Tumour tissue was analysed using semiconductor-based next-generation sequencing technology (Platform – Thermofisher Ion S5; Mapped fusion reads > 20,000; Median read count 120). High-quality tumour tissue RNA extracted from the submitted specimen was subjected to target enrichment by multiplex polymerase chain reaction (PCR) amplification by Sarcoma Fusion panel. Sequenced data was analysed using a customised in-house pipeline DCGL NGS Bioinformatics v4.2. An FDG PET performed on the 17th of July showed no other focus of disease. Hence, the patient was advised to undergo radiotherapy as there is no role for chemotherapy in CCS in the adjuvant setting. In view of delay in flap healing, radiation therapy was delayed and repeat PET scan after 3 months showed metastatic disease to pelvic lymph nodes and lung metastasis. After which he was started on tab pazopanib but died after 2 months.

## Discussion

The above case demonstrates the importance of correctly diagnosing soft tissue tumours in the early phase of presentation itself. CCS has slight female preponderance and is found in 15–35 years age group [12, 14], median age being third decade, similar to our patient. It is most commonly found in the extremities and has a propensity of lymph node metastasis [14].

This case also highlights the pitfalls associated with a delay in referral to a sarcoma speciality clinic in the event of a diagnostic dilemma associated with a STS. Delay in correct diagnosis leads to mistreatment, larger tumour burden, functionally debilitating surgeries and significant distress in the patient which might last longer than treatment duration [15–20]. This study reinforces the need of sarcoma pathologists in developing countries and need for pathology second opinion early in the course of treatment. As per UK guidelines, sarcoma should be reported by sarcoma pathologist who sees significant number of sarcoma in daily practice [21].

A study by Thway *et al* [22] showed that there were 16.4% major discrepancies (those that could lead to significant change in clinical management) and 11.8% minor discrepancies (those in which the discrepancy was not thought to provoke significant management change) in diagnosis of soft tissue neoplasm after referring to a tertiary sarcoma referral unitClick or tap here to enter text.. The major cause of discrepancies appears attributable to differences in interpretation by the referral and tertiary centre pathologist rather than a lack of or inappropriate use of IHC tests at the referring Centre [22]. This justifies the need for specialist referral for soft tissue tumours in a country like Nepal where a discrepancy can be expected to be higher because of both inexperience and lack of facilities.

*EWSR1* gene, located at chromosome22q12, encodes a 656-amino acid nuclear protein that is thought to have roles in meiotic cell division, mitotic spindle formation and stabilisation of microtubules, as well as DNA repair mechanisms and cellular ageing. *EWSR1* is a member of *TET* family of genes which has an RNA binding domain at the carboxy terminus of the gene product which is important in protein-RNA binding, transcription and RNA metabolism [23–25]. *EWSR1* has been identified as a translocation partner in a wide range of clinically and pathologically diverse tumours which include the Ewing family of tumours, desmoplastic small round cell tumour, myxoid liposarcomas, extra-skeletal myxoid chondrosarcoma, angiomatoid fibrous histiocytoma, CCS of soft tissue and clear cell sarcoma-like tumours of the gastrointestinal tract, primary pulmonary myxoid sarcoma, myoepithelial tumours of skin, soft tissue and bone and rare examples of low-grade fibro-myxoid sarcoma, sclerosing epithelioid fibrosarcoma and mesothelioma [25].

The errors that accompanied this patient were inappropriate chemotherapy, as CCS is chemo resistant [26, 27] and the patient could have undergone sentinel node biopsy if the diagnosis was clear earlier [28, 29].

The sensitivity to chemotherapy and immunotherapy in this subset has rarely been demonstrated [27, 30]. However, early diagnosis, surgery, sentinel node biopsy and radiation when required are the current treatment of choice for localised CCS [27].

Our case depicts that the molecular markers should be interpreted in the context of meticulous histopathological examination. Also, in Low-middle income countries (LMICs), a well-structured framework is direly needed to improve sarcoma care in the absence of expert pathology, radiology, dedicated sarcoma surgeons and sarcoma medical oncology. The development of pathology services in LMICs faces a number of challenges, including insufficient funds, lack of skilled personnel at all levels (technicians to pathologists), unavailable equipment and unreliable supply chains for consumables [31]. Patients with sarcoma should be referred to tertiary care centres with established sarcoma teams and patient advocacy should be encouraged to educate and empower patients. This also calls for a second opinion for sarcoma pathology, which should be a well-established dogma, unlike other common cancers.

This case shows how a misdiagnosis can change the entire course of the disease rather appallingly. Discrepancy of pathological diagnosis is a well-established entity as far as STSs are concerned. This case adds to the existing knowledge and given the dire need of expertise in pathology in sarcoma, this needs to be re-emphasised with more cases.

## Conclusion

This case reveals that expert pathology and appropriate molecular tests for rare sarcomas are essential for appropriate management, along with a second pathology opinion for sarcomas at an expert centre, which should be the norm rather than exception.

## Conflicts of interest

No conflicts of interest.

## Funding

We did not receive any funding for this manuscript and have no financial interests.
